# Two-way free-space optics-based interface between fibre and 5G communication employing polarisation-orthogonal modulation

**DOI:** 10.1038/s44172-023-00148-2

**Published:** 2023-12-12

**Authors:** Hai-Han Lu, Xu-Hong Huang, Chung-Yi Li, Yan-Zhen Xu, Jia-Lian Jin, Wei-Xiang Chen, Chih-Hong Lin, Tsai-Man Wu

**Affiliations:** 1 Institute of Electro-Optical Engineering, National Taipei University of Technology, Taipei, 10608 Taiwan; 2 The School of Information Science and Engineering, Fujian University of Technology, 350118 Fujian, China; 3 Department of Communication Engineering, National Taipei University, New Taipei City, 23741 Taiwan

**Keywords:** Microwave photonics, Fibre optics and optical communications

## Abstract

Fifth-generation (5G) communication provides a substantial increase in data transmission capacity because of more available bandwidth and advanced communication techniques. It opens the door to a range of new applications, such as 4K/8K video streaming, Internet of Things, autonomous vehicles, unmanned aerial vehicles, and more. However, atmospheric attenuation poses a technical challenge for 5G communication, especially at higher frequencies. Free-space optics (FSO)-based interface between fibre and 5G communication integrates fibre optics, FSO and 5G communications, achieving high-speed and long-distance transmission and providing an alternative for 5G signal transport. Here we report a two-way fibre-FSO-5G wireless communication employing polarisation-orthogonal modulation. After 25-km single-mode fibre, 1-km FSO, and 20-m (millimetre-wave)/10-m (sub-6 GHz) radio-frequency wireless transport, satisfactorily low bit error rates and error vector magnitudes are acquired, meeting the demands of 5G emerging applications.

## Abstract (web summary)

Lu and coauthors use two orthogonal polarisations to separate downstream and upstream data flows in connected fibre-free-space optics-5G wireless communication. They achieve an undisruptive signal transmission and enable a high-throughput interface.

## Introduction

**Fig. 1:**
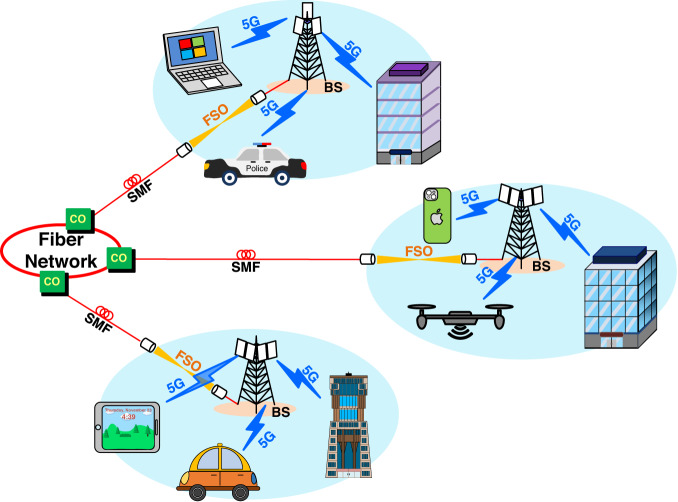
5G (5G, fifth-generation) signals through fibre-FSO (FSO, free-space optics)-wireless communications. The development of 5G signals through fibre-FSO-wireless communications has greatly accelerated the global trend. CO central office, SMF single-mode fibre, BS base station.

High data rate, ultra-low latency, and massive connection requirements in fifth-generation (5G) services have substantially promoted the development of telecommunications^1–8^. The development of 5G signals through fibre-free-space optics (FSO)-wireless communications (as shown in Fig. 1) has greatly accelerated this global trend. One of the technical challenges of 5G wireless communications is high atmospheric attenuation. 5G communication experiences severe signal loss as 5G signals propagate through the atmosphere, limiting their coverage. Reducing high atmospheric attenuation is a substantial challenge in deploying 5 G communication. A promising solution to address this limitation is the deployment of FSO links. It overcomes the coverage limitations of 5G signal transmission. FSO links are expected to compensate for the atmospheric loss of 5G signal transmission and considerably reduce the number of 5G base stations^9–13^. Integrating fibre optics, FSO, and 5G communications, the FSO-based interface between fibre and 5G communication enables high-speed and long-distance transmission. A two-way fibre–FSO–5G wireless communication was previously demonstrated employing single-carrier optical modulation for downlink transmission and phase modulation (PM) for uplink transmission^14^. Furthermore, former work showed a two-way fibre–FSO–5G new radio communication using intensity modulation (IM) for downlink transmission and PM for uplink transmission^15^. However, this two-way fibre–FSO–5G wireless communication system faces some challenges, particularly related to the complicated optical frequency comb source and the sophisticated PM-to-IM conversion with injection-locked distributed feedback (DFB) laser diode (LD). Developing a low-complexity configuration is crucial for the successful deployment of two-way fibre–FSO–5G wireless communication systems. In addition, with the IM-to-PM effect, the downstream intensity-modulated signal with a high optical modulation index introduces distortions that degrade the upstream transmission performance. To maintain acceptable upstream transmission performance, it is necessary to reduce the optical modulation index of the downstream intensity-modulated signal, which can lead to a degraded downstream performance^16,17^. This trade-off between the optical modulation index and downstream/upstream performance needs to be carefully managed to optimise the system performance.

In this demonstration, we report the deployment of two-way fibre–FSO–5G wireless communications using polarisation-orthogonal modulation. It is understood that this is the first successful construction of a two-way FSO-based interface between fibre and 5G communication with polarisation-orthogonal modulation. By implementing polarisation-orthogonal modulation, the polarisation of the central carrier (*x*-polarisation) is orthogonal with the optical sidebands modulated with 5G millimetre-wave (MMW) and sub-6 GHz signals (*y*-polarisation). It shows a 5G communication system with a low-complexity configuration to split the central carrier for the upstream carrier and optical sidebands for downstream transmission. The use of the polarisation-orthogonal modulation technique reduces the complexity of the system, eliminating the need for a complicated optical frequency comb source and sophisticated PM-to-IM conversion with remotely injection-locked DFB LD. It holds the potential for achieving long-distance 5G MMW/sub-6 GHz communications with high transmission rates. The light source, containing a DFB LD, a polarisation rotator (PR) and a Mach–Zehnder modulator (MZM), is utilized as an alternative to an optical frequency comb source to simplify the configuration. The downstream light is rotated through a PR and modulated by an MZM with 16-quadrature amplitude modulation (QAM)-orthogonal frequency-division multiplexing (OFDM) signals. The MZM is biased at the minimum transmission point, which results in *y*-polarised downstream light being modulated in an optical carrier suppression form^18,19^. Meanwhile, the *x*-polarised unmodulated carrier is picked using a polarisation beam splitter at the downstream reception site and reused for the upstream carrier. The upstream carrier is modulated by an MZM with 16-QAM-OFDM signals as well. We bias the MZM at the minimum transmission point, which leads to the *x*-polarised upstream light being modulated in an optical carrier suppression form. The downstream *y*-polarised (upstream *x*-polarised) signal is optically converted from a radio-frequency (RF) signal with low frequency to a 5G MMW/sub-6 GHz signal with high frequency. By operating in the 5G MMW/sub-6 GHz band, the system can carry more data due to the increased carrier frequency^20–22^.

A two-way fibre–FSO–5G wireless communication system employing polarisation-orthogonal modulation is offered and realised. For downstream transmission, *y*-polarised intensity-modulated 10-Gbps/38-GHz and 1-Gbps/4.4-GHz 16-QAM-OFDM signals through fibre–FSO–5G wireless communication is practically built. For upstream transmission, *x*-polarised intensity-modulated 10-Gbps/26-GHz and 1-Gbps/3.7-GHz 16-QAM-OFDM signals transport through FSO–fibre–5G wireless communication is practically constructed. 3.7 and 4.4 GHz carriers are adopted for 5G signal transmission to meet 5G sub-6 GHz frequency band (410 MHz–7.125 GHz) demands, and 26 and 38 GHz carriers are adopted for 5G signal transmission to satisfy 5G MMW frequency band (24.25–71 GHz) requirements. With an in-depth observation of a two-way fibre–FSO–5G wireless communication system, good performance of low bit error rate (BER) (<3.8 × 10^−3^ forward error correction threshold) and error vector magnitude (EVM) (<12.5% third-generation partnership project (3GPP) limit)^23,24^, as well as clear and distinct constellation diagrams are achieved through a distance of 25 km single-mode fibre (SMF), 1 km FSO, and 20 m/10 m 5G wireless. The successful establishment of the two-way FSO-based interface between fibre and 5G communication marks a crucial step in the implementation and development of 5G MMW/sub-6 GHz communications. It has a major impact on the integration of fibre optics, FSO, and MMW/sub-6 GHz communications and the use of employing polarisation-orthogonal modulation. It can serve as a reference model for further enhancement and future development of 5G communications.

## Results and discussion

### Atmospheric attenuation and laser light alignment for FSO links

When optical signals travel through free space, atmospheric turbulence causes the optical signals to attenuate in the atmosphere. For FSO links, atmospheric attenuation varies with weather conditions. Over a 1-km FSO link, atmospheric attenuation changes from 2 dB (clear weather) to 50 dB (bad weather). Severe atmospheric turbulence caused by severe weather strongly affects FSO link performance. In this demonstration, approximately 2.6 dB of atmospheric attenuation occurs (clear weather) due to a 1 km FSO link. However, under severe weather conditions such as heavy rain and fog, FSO link performance will drastically deteriorate. In severe weather, nevertheless, a modified new zero-cross correlation code can be used to enhance FSO link performance^25^. Furthermore, laser light alignment is a critical issue for FSO links as it directly affects link performance and reliability. Laser light misalignment can cause performance degradation, making the FSO link unreliable. For laser light alignment, the key issue is to entirely direct the laser light into the input of fibre ferrule at the reception site. When the laser light transmits over a 1-km FSO link, it is quite challenging to entirely direct the laser light into the fibre ferrule input thanks to the natural features of laser spreading. A reducing device should be deployed to reduce the size of the laser beam so that the laser light can be entirely directed into the input of fibre ferrule. The doublet lens at the reception site is a reducing device that reduces the size of the laser beam and precisely directs it into the fibre ferrule input.

### Downstream/upstream performance degradation due to RF fading, equalisation-enhanced phase noise, and self-polarisation/cross-polarisation interferences

For multiple optical carrier schemes, RF fading due to fibre dispersion and equalisation enhanced phase noise due to interaction between laser phase noise and electronic dispersion compensation module reduce system performance, resulting in worse downstream/upstream transmission performance^26^. However, since the SMF is only 25 km in length and is a short-distance communication system rather than a long-haul communication system, the impact of RF fading and equalisation-enhanced phase noise on the downstream/upstream performance degradation is limited. In addition, in scenarios where both downstream and upstream signals are transported through the same SMF, self-polarisation/cross-polarisation interferences due to optical beating can be minimised by utilizing an optical band-pass filter and a polarisation-orthogonal modulation technique in the system. After 25-km SMF with 1-km FSO transmissions (downstream)/1-km FSO with 25-km SMF transmissions (upstream), using the optical band-pass filter at the reception site can drastically filter out self-polarisation interferences, thereby enhancing downstream/upstream performance. Moreover, since *x*-polarised (upstream) and *y*-polarised (downstream) sidebands are orthogonal, no cross-polarisation interferences exist, thereby improving downstream/upstream performance^27^.

### Downstream/upstream BERs/EVMs at different received MMW/sub-6 GHz powers over 25-km SMF, 1-km FSO, and 20-m/10-m RF wireless cascaded-medium

**Fig. 2:**
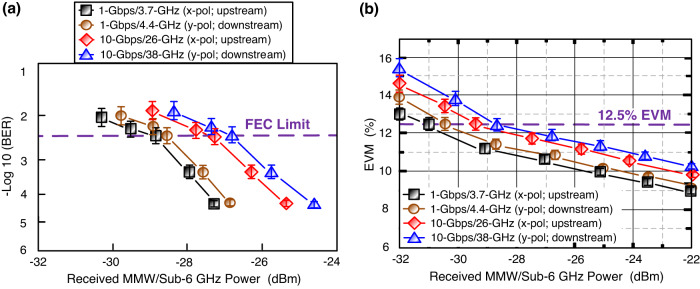
Downstream/upstream BERs (BERs, bit error rates)/EVMs (EVMs, error vector magnitudes). Downstream/upstream **a** BERs and **b** EVMs at different received MMW (MMW, millimetre-wave)/sub-6 GHz powers over 25-km SMF (SMF, single-mode fibre), 1-km FSO (FSO, free-space optics), and 20-m (MMW)/10-m (sub-6 GHz) RF (RF, radio-frequency) wireless cascaded-medium. Black square/line indicates 1-Gbps/3.7-GHz signal (*x*-polarisation; upstream), brown circle/line indicates 1-Gbps/4.4-GHz signal (*y*-polarisation; downstream), red rhombus/line indicates 10-Gbps/26-GHz signal (*x*-polarisation; upstream), and blue triangle/line indicates 10-Gbps/38-GHz signal (*y*-polarisation; downstream). The error bars represent the standard deviations of the measured data from three experimental trials.

Figure 2a shows the downstream/upstream BERs at different received MMW/sub-6 GHz powers over 25-km SMF, 1-km FSO, and 20-m (MMW)/10-m (sub-6 GHz) RF wireless cascaded medium. For 10-Gbps/38-GHz and 1-Gbps/4.4-GHz 16-QAM-OFDM signals transport (*y*-polarisation; downstream), we achieve a 3.8 × 10^−3^ (forward error correction limit) BER at −26.7 and −28.5 dBm received MMW/sub-6 GHz powers, and we attain a 4.7 × 10^−5^ (<3.8 × 10^−3^ forward error correction limit) BER at −24.6 and −26.8 dBm received MMW/sub-6 GHz powers. For 10-Gbps/26-GHz and 1-Gbps/3.7-GHz 16-QAM-OFDM signals transport (*x*-polarisation; upstream), we achieve a BER of 3.8 × 10^−3^ at −27.4 and −28.8 dBm received MMW/sub-6 GHz powers, and we attain a BER of 4.7 × 10^−5^ at −25.4 and −27.4 dBm received MMW/sub-6 GHz powers. Phase noise increases with the increase in carrier frequency^28^. For the 10-Gbps/38-GHz 16-QAM-OFDM signal (downstream), the phase noise is higher compared to the 1-Gbps/4.4-GHz 16-QAM-OFDM signal. For the 10-Gbps/26-GHz 16-QAM-OFDM signal (upstream), the phase noise is higher compared to the 1-Gbps/3.7-GHz 16-QAM-OFDM signal. Higher carrier frequency leads to higher phase noise, which in turn requires higher received power to maintain a desired BER. Under the same BER, the 10-Gbps/38-GHz (10-Gbps/26-GHz) signal requires a higher received power compared to the 1-Gbps/4.4-GHz (1-Gbps/3.7-GHz) signal. To achieve the same BER, the signal transmitted at higher carrier frequency requires more received power to compensate for the decline in signal-to-noise ratio (SNR) and maintain a specific *Q*-factor due to the impact of higher phase noise associated with higher carrier frequency. Therefore, compensating for a drop in SNR means compensating for a drop in *Q*-factor as well. In addition, the measured EVMs at different received MMW/sub-6 GHz powers are exhibited in Fig. 2b. Over 25-km SMF, 1-km FSO, and 20-m (MMW)/10-m (sub-6 GHz) 5G wireless transports, the EVMs of four 16-QAM-OFDM signals are less than the 12.5% 3GPP limit as the received MMW/sub-6 GHz powers are higher than −28.7 (*y*-polarised 10-Gbps/38-GHz; downstream), −29.4 (*x*-polarised 10-Gbps/26-GHz; upstream), −30.5 (*y*-polarised 1-Gbps/4.4-GHz; downstream), and −31 (*x*-polarised 1-Gbps/3.7-GHz; upstream) dBm, respectively. Since EVM is directly associated with EVM_WN_ (EVM induced by white noise) and EVM_PhN_ (EVM induced by phase noise), yet lower EVM_WN_ and lower EVM_PhN_ contribute to lower EVM^29,30^. Given that the EVM_WN_ is directly associated with the peak-to-average power ratio, *y*-polarised 1-Gbps/4.4-GHz and *x*-polarised 1-Gbps/3.7-GHz 16-QAM-OFDM signals with lower carrier frequencies have lower peak-to-average power ratios, bringing on lower EVM_WN_. In addition, given that the EVM_PhN_ is directly related to the carrier frequency, *y*-polarised 1-Gbps/4.4-GHz and *x*-polarised 1-Gbps/3.7-GHz 16-QAM-OFDM signals with lower carrier frequencies have less phase noise, bringing on lower EVM_PhN_. Due to lower EVM_WN_ and EVM_PhN_, the *y*-polarised 1-Gbps/4.4-GHz and *x*-polarised 1-Gbps/3.7-GHz 16-QAM-OFDM signals exhibit lower overall EVM at the same received power. To achieve the same EVM, the *y*-polarised 10-Gbps/38-GHz and *x*-polarised 10-Gbps/26-GHz 16-QAM-OFDM signals experience higher received power to compensate for the decline in SNR and maintain a specific *Q*-factor due to EVM_WN_ and EVM_PhN_ performance degradation.

### The connected constellation diagrams and eye patterns of 10-Gbps/38-GHz and 1-Gbps/4.4-GHz (*y*-polarisation; downstream), as well as 10-Gbps/26-GHz and 1-Gbps/3.7-GHz (*x*-polarisation; upstream) 16-QAM-OFDM signals

**Fig. 3:**
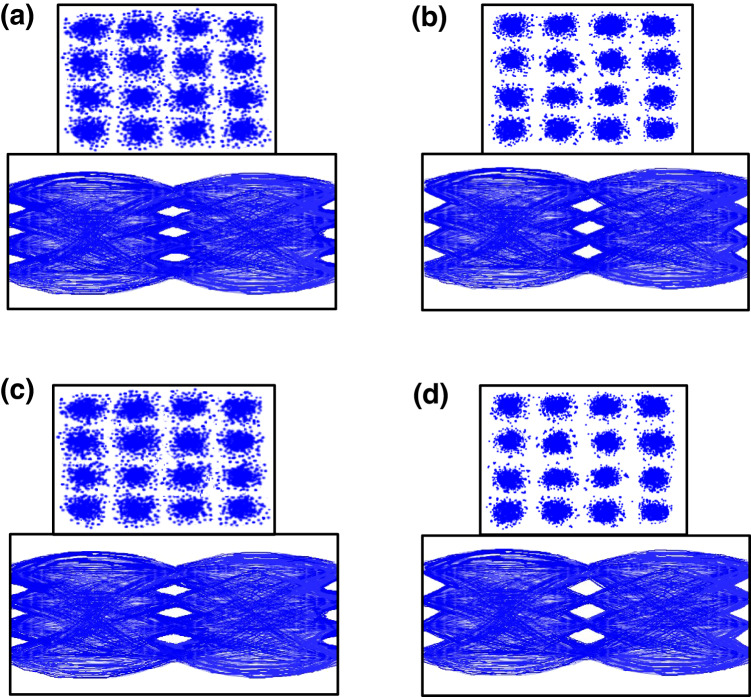
The connected constellation diagrams and eye patterns of 10-Gbps/38-GHz and 1-Gbps/4.4-GHz (*y*-polarisation; downstream), as well as 10-Gbps/26-GHz and 1-Gbps/3.7-GHz (*x*-polarisation; upstream) signals. The associated constellation diagrams and eye patterns (in-phase component) of **a** 10-Gbps/38-GHz and **b** 1-Gbps/4.4-GHz 16-QAM-OFDM (16-QAM-OFDM, 16-quadrature amplitude modulation-orthogonal frequency-division multiplexing) signals (*y*-polarisation; downstream) at 3.8 × 10^−3^ BER (BER, bit error rate). The correlated constellation diagrams and eye patterns (in-phase component) of **c** 10-Gbps/26-GHz and **d** 1-Gbps/3.7-GHz 16-QAM-OFDM signals (*x*-polarisation; upstream) at 4.7 × 10^−5^ BER.

Figures 3a, b exhibit the associated constellation diagrams and eye patterns (in-phase component) of 10-Gbps/38-GHz and 1-Gbps/4.4-GHz 16-QAM-OFDM signals (*y*-polarisation; downstream) at 3.8 × 10^−3^ BER. For 10-Gbps/38-GHz 16-QAM-OFDM signal transport, clear constellation diagrams and eye patterns (Fig. 3a) are acquired at −26.7 dBm received MMW power. For 1-Gbps/4.4-GHz 16-QAM-OFDM signal transmission, clear and distinct constellation diagrams and eye patterns (Fig. 3b) are acquired at −28.5 dBm received sub-6 GHz power. Furthermore, Fig. 3c, d show the correlated constellation diagrams and eye patterns (in-phase component) of 10-Gbps/26-GHz and 1-Gbps/3.7-GHz 16-QAM-OFDM signals (*x*-polarisation; upstream) at 4.7 × 10^−5^ BER. For 10-Gbps/26-GHz 16-QAM-OFDM signal transport, clear constellation diagrams and eye patterns (Fig. 3c) are acquired at a received MMW power of −25.4 dBm. For 1-Gbps/3.7-GHz 16-QAM-OFDM signal transmission, very clear and distinct constellation diagrams and eye patterns (Fig. 3d) are obtained at a received sub-6 GHz power of −27.4 dBm. The clarity and distinctness of the constellation diagrams and eye patterns show that this demonstrated two-way fibre–FSO–wireless communication system is practicable for transporting 5G signals over both MMW and sub-6 GHz frequencies.

### The subcarrier EVMs of the 10-Gbps/26-GHz (*x*-polarisation; upstream), 10-Gbps/38-GHz (*y*-polarisation; downstream), 1-Gbps/3.7-GHz (*x*-polarisation; upstream), and 1-Gbps/4.4-GHz (*y*-polarisation; downstream) 16-QAM-OFDM signals at different subcarrier indices

**Fig. 4:**
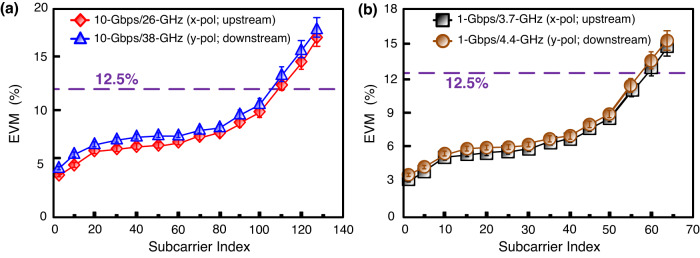
The subcarrier EVMs (EVMs, error vector magnitudes) at different subcarrier indices. The subcarrier EVMs of **a** 10-Gbps/26-GHz (*x*-polarisation; upstream) and 10-Gbps/38-GHz (*y*-polarisation; downstream), and **b** 1-Gbps/3.7-GHz (*x*-polarisation; upstream) and 1-Gbps/4.4-GHz (*y*-polarisation; downstream) 16-QAM-OFDM (16-QAM-OFDM, 16-quadrature amplitude modulation-orthogonal frequency-division multiplexing) signals at different subcarrier indices. Red rhombus/line indicates 10-Gbps/26-GHz signal (*x*-polarisation; upstream), blue triangle/line indicates 10-Gbps/38-GHz signal (*y*-polarisation; downstream), black square/line indicates 1-Gbps/3.7-GHz signal (*x*-polarisation; upstream), and brown circle/line indicates 1-Gbps/4.4-GHz signal (*y*-polarisation; downstream). The error bars represent the standard deviations of the measured data from three experimental trials.

Figure 4a shows the subcarrier EVMs of the 10-Gbps/26-GHz (*x*-polarisation; upstream) and 10-Gbps/38-GHz (*y*-polarisation; downstream) 16-QAM-OFDM signals at different subcarrier indices. Over 25-km SMF, 1-km FSO, and 20-m 5G wireless hybrid-medium, the EVMs of two downstream/upstream 16-QAM-OFDM signals are less than the 12.5% 3GPP limit as the subcarrier indices are lower than 109 (10-Gbps/26-GHz) and 105 (10-Gbps/38-GHz), respectively. Moreover, Fig. 4b presents the subcarrier EVMs of the 1-Gbps/3.7-GHz (*x*-polarisation; upstream) and 1-Gbps/4.4-GHz (*y*-polarisation; downstream) 16-QAM-OFDM signals at different subcarrier indices. Over 25-km SMF, 1-km FSO, and 10-m 5G wireless cross-medium, the EVMs of two downstream/upstream 16-QAM-OFDM signals are lower than the 12.5% 3GPP requirement as the subcarrier indices are lower than 58 (1-Gbps/3.7-GHz) and 56 (1-Gbps/4.4-GHz), respectively. Additionally, it is to be noted that there are positive slopes for the EVMs. EVMs have low values at low subcarrier index and are proportional to the subcarrier index. The average EVMs are around 8.2% (10-Gbps/26-GHz), 8.4% (10-Gbps/38-GHz), 7.2% (1-Gbps/3.7-GHz), and 7.3% (1-Gbps/4.4-GHz), which are less than the 12.5% required by the 3GPP limit. Low average EVM show the achievability of constructing a two-way fibre–FSO–5G wireless communication system using polarisation-orthogonal modulation for both downstream and upstream transmissions.

## Methods

### 5G MMW/sub-6 GHz signals through two-way fibre–FSO–wireless communications

**Fig. 5:**
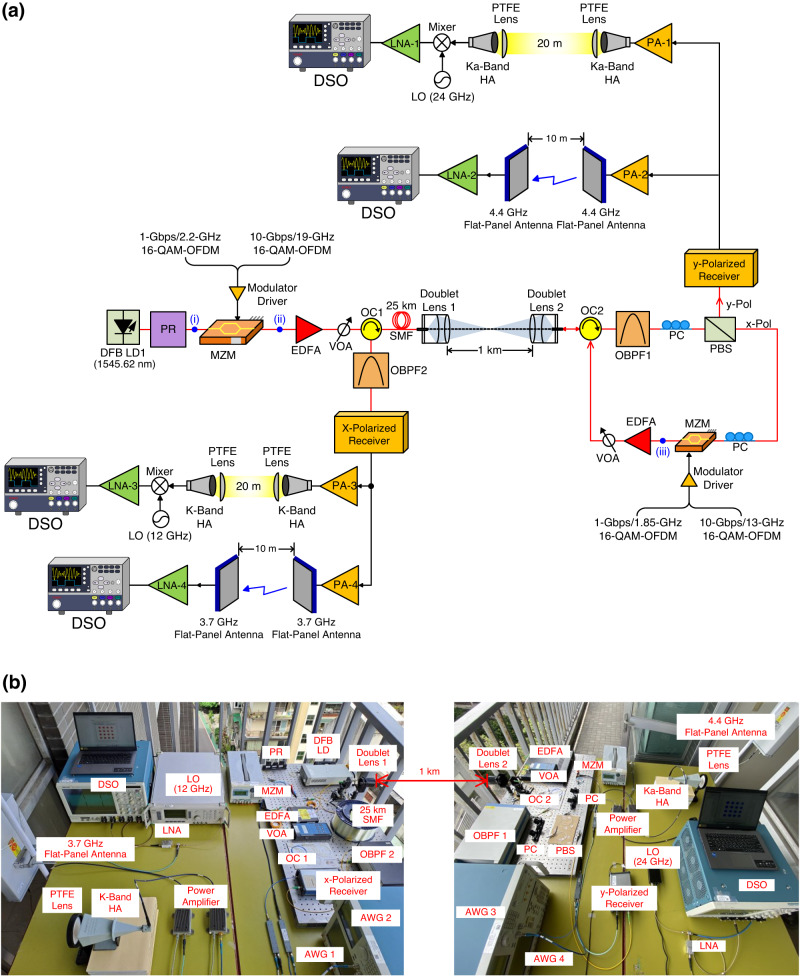
5G (5G, fifth-generation) MMW (MMW, millimetre-wave)/sub-6 GHz signals through two-way fibre–FSO (FSO, free-space optics)-wireless communications. **a** Architecture of 5G MMW/sub-6 GHz signals through two-way fibre–FSO–wireless communications employing polarisation-orthogonal modulation technique. **b** A photo of experimental setup. DFB LD distributed feedback laser diode, PR polarisation rotator, 16-QAM-OFDM 16-quadrature amplitude modulation-orthogonal frequency-division multiplexing, MZM Mach–Zehnder modulator, EDFA erbium-doped fibre amplifier, VOA variable optical attenuator, OC optical circulator, SMF single-mode fibre, OBPF optical band-pass filter, PC polarisation controller, PBS polarisation beam splitter, PA power amplifier, PTFE polytetrafluoroethylene, HA horn antenna, LO local oscillator, LNA low noise amplifier, DSO digital sampling oscilloscope.

**Fig. 6:**
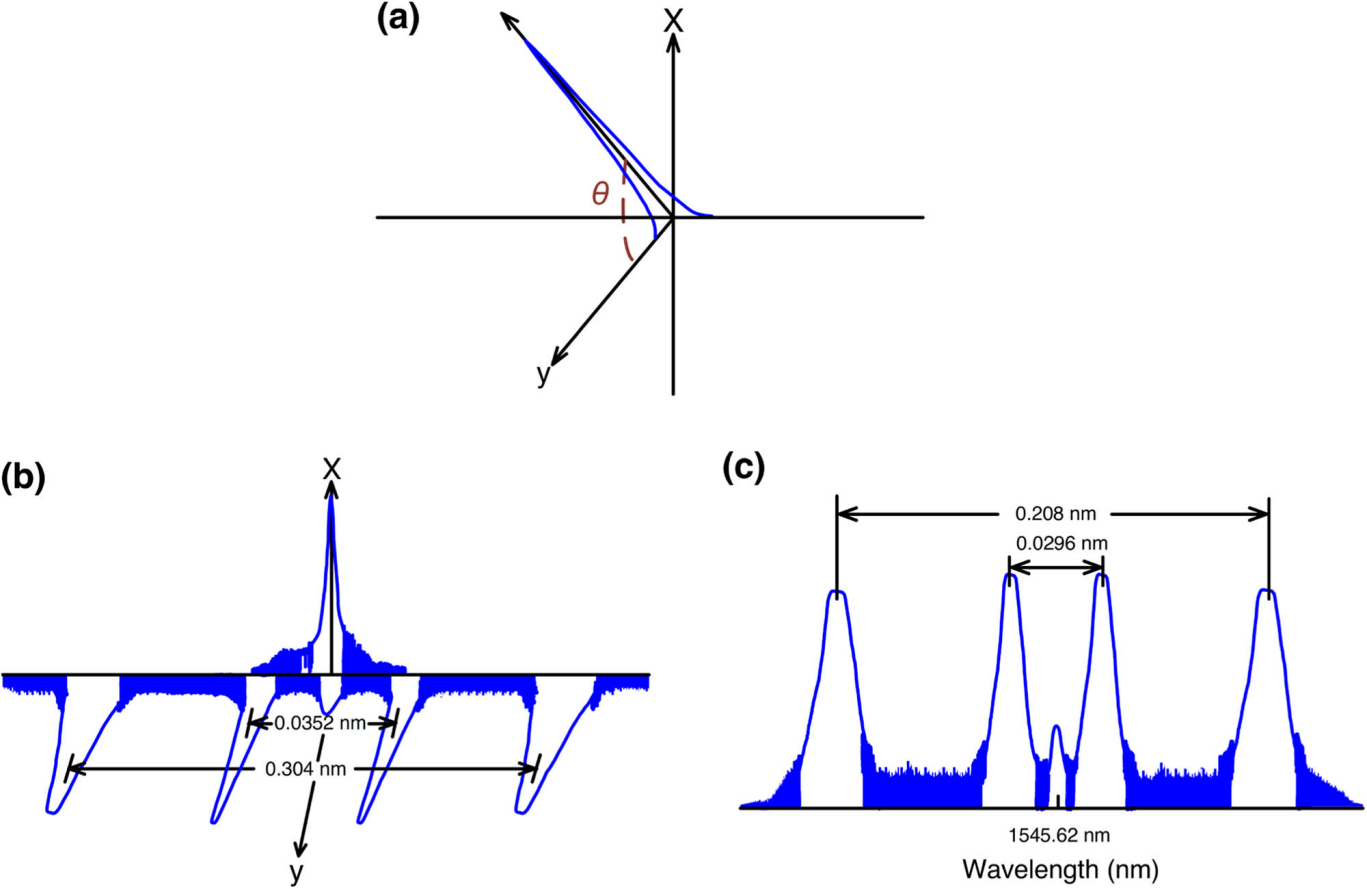
The optical spectrum at different points of Fig. 5a. **a** The optical spectrum behind the PR (PR, polarisation rotator) (inset (i) of Fig. 5a). **b** The optical spectrum behind the MZM (MZM, Mach–Zehnder modulator) (downstream transmission site) (inset (ii) of Fig. 5a). **c** The optical spectrum after the MZM (upstream transmission site) (inset (iii) of Fig. 5a).

The architecture of 5G MMW/sub-6 GHz signals through two-way fibre–FSO–wireless communications employing polarisation-orthogonal modulation is offered and realised in Fig. 5a. A real experimental setup rather than a simulated one is established. And further, a photo of the experimental setup is exhibited in Fig. 5b. A light source, including a DFB LD, a PR and an MZM, is deployed at the transmission site. The light sent out from a DFB LD (with 1545.62 nm centre wavelength) is supplied to an MZM through a PR. The MZM is worked at the minimum transmission point and is driven by integrated 1-Gbps/2.2-GHz and 10-Gbps/19-GHz 16-QAM-OFDM signals through the modulator driver. PR rotates the polarisation direction of polarised light by *θ* angle. The optical spectrum behind the PR is given in Fig. 6a (inset (i) of Fig. 5a). Due to the electro-optical characteristics of LiNbO_3_ crystal, the half-wave voltage *V*_*π*_ in the *x*-direction is ~3.58 times larger than that in the *y*-direction. Owing to higher *V*_*π*_ in the *x*-direction, the *y*-polarised light is effectively modulated as it passes through the LiNbO_3_ crystal. The *x*-polarised light, on the other hand, remains unmodulated^31^. Since the MZM is biased at the minimum transmission point, *y*-polarised light is modulated in the form of optical carrier suppression. The *y*-polarised downstream sidebands are thus optically converted from a 1-Gbps/2.2-GHz signal to 1-Gbps/4.4-GHz 5G sub-6 GHz signal, and from a 10-Gbps/19-GHz signal to 10-Gbps/38-GHz 5G MMW signal. Whereas the *x*-polarised light is transmitted and reused as an upstream carrier. The optical spectrum behind the MZM is given in Fig. 6b (inset (ii) of Fig. 5a). Clearly, the wavelength separations of the ±1 and ±2 sidebands are 0.0352 nm (4.4 GHz) and 0.304 nm (38 GHz), respectively.

After IM, the optical signal is magnified by an erbium-doped filter amplifier (EDFA). The function of EDFA is to amplify the optical signal to compensate for the 25 km SMF transmission loss (~6.4 dB) and 1 km FSO link loss (~2.6 dB). A variable optical attenuator is introduced after the EDFA to reduce distortion as the optical power supplied to the fibre is reduced. The optical signal is then delivered by a 25-km SMF with 1-km FSO transmissions via two optical circulators (OCs; OC1 and OC2) and a set of doublet lenses with 50.8 mm diameter and 150 mm focal length. A 1-km FSO link is established between two buildings to transport laser light to the free space. Regarding the energy consumption of a 5G communication system, a 1-km FSO link can considerably reduce the need for a large number of 5G base stations. As the number of 5G base stations decreases, the number of power amplifiers (PAs) required to amplify signals also decreases. PAs are known as the most power-consuming components in 5G communication systems. The decrease in the number of PAs results in a substantial decrease in energy consumption. Through a 1-km FSO link, the downstream optical signal is circulated by an OC, filtered by an optical band-pass filter, split by a polarisation controller with a polarisation beam splitter, and received by a *y*-polarised receiver. After split by an RF splitter, the signal passes through two independent PAs. For the upper part, a PA (PA-1) amplifies the *y*-polarised 10-Gbps/38-GHz 5G MMW signal. PA-1 with 37.5–42.5 GHz ranges has the capability to amplify a desirable 10-Gbps/38-GHz signal while simultaneously filter out an undesirable 1-Gbps/4.4-GHz signal. The amplified signal is then transmitted wirelessly by a set of horn antennas (HAs) operating in Ka-band at 26.5–40 GHz. The MMW beam is radiated to free space through a set of polytetrafluoroethylene (PTFE) lenses operating at MMW frequencies^32,33^. After wireless transmission at 20 m distance, the 10-Gbps/38-GHz signal is down-converted to the 10-Gbps/14-GHz intermediate frequency signal through a mixer with a 24-GHz local oscillator signal. Afterwards, the down-converted signal is enhanced by a low noise amplifier (LNA-1) in a range of 6–20 GHz and sent to a digital sampling oscilloscope for downstream performance evaluation. For the lower part, a PA (PA-2) with 4.4–4.9 GHz ranges amplifies the *y*-polarised 1-Gbps/4.4-GHz 5G sub-6 GHz signal. PA-2 with 4.4–4.9 GHz ranges has the capability to boost a desirable 1-Gbps/4.4-GHz signal while concurrently removing an undesirable 10-Gbps/38-GHz signal. Next, a couple of flat-panel antennas with 4.4–5.1 GHz ranges and 15 dBi gain wirelessly transport it. Afterwards, the 1-Gbps/4.4-GHz 5G sub-6 GHz signal is promoted by an LNA (LNA-2) with 2–6 GHz ranges and sent to a digital sampling oscilloscope for downstream performance analysis.

For upstream, the *x*-polarised central optical carrier split by the polarisation beam splitter is reused and modulated by an MZM with integrated 1-Gbps/1.85-GHz and 10-Gbps/13-GHz 16-QAM-OFDM signals. The MZM is worked at the minimum transmission point as well, which leads to the *x*-polarised upstream light being modulated in an optical carrier suppression form. The *x*-polarised upstream sidebands are therefore optically converted from a 1-Gbps/1.85-GHz signal to 1-Gbps/3.7-GHz 5G sub-6 GHz signal, and from a 10-Gbps/13-GHz signal to 10-Gbps/26-GHz 5G MMW signal. The optical spectrum after the MZM is presented in Fig. 6c (inset (iii) of Fig. 5a). Apparently, the wavelength separations of the ±1 and ±2 sidebands are 0.0296 nm (3.7 GHz) and 0.208 nm (26 GHz), respectively. The EDFA then amplifies the optical signal, the variable optical attenuator controls it, and the OC2 circulates it. Over 1-km FSO and 25-km SMF links, the upstream optical signal is bridged by an OC, filtered by an optical band-pass filter, and received by an *x*-polarised receiver. After splitting, the signal travels through two independent PAs. For the upper path, a PA (PA-3) boosts the *x*-polarised 10-Gbps/26-GHz 5G MMW signal. PA-3 with 26–28 GHz ranges has the ability to boost a desirable 10-Gbps/26-GHz signal while simultaneously attenuating an undesirable 1-Gbps/3.7-GHz signal. The boosted signal is then transported wirelessly by a couple of HAs operating in K-band at 18–26.5 GHz. The MMW beam is radiated to free space through a pair of PTFE lenses operating at MMW frequencies. Over 20-m RF wireless transport, we down-convert a 10-Gbps/26-GHz signal into a 10-Gbps/14-GHz signal through a mixer with a 12-GHz local oscillator signal. Subsequently, the down-converted signal is enhanced by an LNA (LNA-3) in a range of 6–20 GHz and sent to a digital sampling oscilloscope for upstream performance evaluation. For the lower path, a PA (PA-4) amplifies the *x*-polarised 1-Gbps/3.7-GHz 5G sub-6 GHz signal. PA-4 with 3.3–3.8 GHz ranges has the natural characteristics to promote the 1-Gbps/3.7-GHz signal and concurrently eliminate the 10-Gbps/26-GHz signal. Next, a couple of flat-panel antennas with 3.3–3.8 GHz ranges and 17 dBi gain wirelessly transmit it. After enhancing by an LNA (LNA-4) with 2–6 GHz ranges, a digital sampling oscilloscope receives the enhanced signal.

For better understanding, a table (Table 1) outlines the downstream/upstream optical power level at key points within the setup, such as the output of MZM, the output of EDFA, the input and output of 25 km SMF, and the input and output of 1 km FSO link. In addition, Table 2 lists and describes the physical parameters of the two-way FSO-based interface between fibre and 5 G communication, including fibre optics, FSO, and 5G wireless.

**Table 1:** The optical power level at key points within the setup.

Downstream	Key points within the setup	Output of MZM	Output of EDFA	Input of 25 km SMF	Output of 25 km SMF	Output of 1 km FSO Link
	Optical power level (dBm)	−3.2	16.2	12.2	5.8	3.2
Upstream	Key points within the setup	Output of MZM	Output of EDFA	Input of 1 km FSO Link	Output of 1 km FSO Link	Output of 25 km SMF
	Optical power level (dBm)	−3.4	16.1	12.2	9.6	3.2

The downstream/upstream optical power level at key points within the setup, such as the output of MZM (MZM, Mach–Zehnder modulator), the output of EDFA (EDFA, erbium-doped fibre amplifier), the input and output of 25 km SMF (SMF, single-mode fibre), and the input and output of 1 km FSO (FSO, free-space optics) link.

**Table 2:** The physical parameters of the two-way communication.

Parameter	Fibre	FSO	5G Wireless		
Descriptions	• 1545.62 nm (DFB LD centre wavelength) • 25 km SMF • 6 dBm transmission loss	• 1 km FSO Link • 2.2 dB Link Loss • Doublet Lens concave lens + convex lens 50.8 mm (diameter) 150 mm (focal length)	Signal format	HA	PTFE Lens
			Downstream • 10-Gbps/38-GHz 5 G MMW • 1-Gbps/4.4-GHz 5G Sub-6 GHz	Downstream • 26.5–40 GHz 25 dBi gain Ka-band HA • 4.4-5.1 GHz 15 dBi gain flat-panel antenna	• 4” (diameter) • 151.5 mm (focal length)
			Upstream • 10-Gbps/26-GHz 5 G MMW • 1-Gbps/3.7-GHz 5 G Sub-6 GHz	Upstream • 18–26.5 GHz 20 dBi gain K-band HA • 3.3–3.8 GHz 17 dBi gain flat-panel antenna	

The physical parameters of the two-way FSO (FSO, free-space optics)-based interface between fibre and 5G (5G, fifth-generation) communication, including fibre optics, FSO, and 5G wireless. DFB LD distributed feedback laser diode, SMF single-mode fibre, HA horn antenna, PTFE polytetrafluoroethylene.

As for synchronization, frame synchronization can be applied in this two-way fibre–FSO–wireless communication system to enhance the overall system’s performance^34^. Data can be divided into frames and synchronization patterns are contained within these frames. The receiver can detect these patterns to align them with the transmitter’s frame structure.

### The integration of HAs and PTFE lenses for 5G MMW signal transmission

For 38-GHz 5G MMW signal transmission, the transmission power before the Ka-band HA is integrated with the PTFE lens and the reception power after the PTFE lens is integrated with the Ka-band HA is measured. A transmission power of 6.2 dBm and a reception power of −24.2 dBm are attained. For HAs integrated with PTFE lenses, the reception power (*P*_R_) can be calculated using Friis’s equation^35,36^:

1 $${P}_{{{{{{\rm{R}}}}}}}={P}_{{{{{{\rm{T}}}}}}}+{G}_{{{{{{\rm{T}}}}}}}+{G}_{{{{{{\rm{R}}}}}}}-20\log \left(4\pi {df} \! / \!c\right)$$

where *P*_T_ is the transmission power, *G*_T_ is the gain of HA integrated with PTFE lens at the wireless transmission site, *G*_R_ is the gain of PTFE lens integrated with HA at the wireless reception site, *d* is the wireless transmission distance, *f* is the carrier frequency, *c* is the speed of light, and *A* is the atmospheric loss. For Ka-band HAs integrated with PTFE lenses, *P*_*T*_ is 6.2 dBm, *G*_T_ and *G*_R_ are 36 dBi, and *d* is 20 m. When the carrier frequency is 38 GHz, the atmospheric loss *A* for 20 m wireless transmission is approximately 10.6 dB. The 20 log(4*πdf/c)* (20 log(4*π*20(38 × 10^9^)/(3 × 10^8^))) is calculated as 90.1 dB, and the reception power is thus calculated as −22.5 (6.2 + 36 + 36–90.1–10.6) dBm. The −22.5 dBm calculated reception power is close to the −24.2 dBm measured reception power. Analysis demonstrates the feasibility of using Ka-band HAs integrated with PTFE lenses for long-distance wireless transmission up to 20 m. In addition, the analysis indicates that the input power of the LNA is far lower than its input 1 dB compression point (10 dBm). It means that the LNA is operating within its linear region.

### Supplementary information

Description of Additional Supplementary Files

Supplementary Data 1

Supplementary Data 2

Supplementary Data 3

Supplementary Data 4

## Data Availability

The data in this manuscript are available from the corresponding author upon reasonable request. The source data for Figs. 2a, b, 4a, and b are provided as Supplementary Data 1–4, respectively.
