# Supplementary material for: Two-way free-space optics-based interface between fibre and 5G communication employing polarisation-orthogonal modulation
Source: Commun Eng. 2023 Dec 12;2:89. doi: 10.1038/s44172-023-00148-2 (PMC10955964; doi:10.1038/s44172-023-00148-2)
Supplement: Supplementary file 1 — Description of Additional Supplementary Files [file 44172_2023_148_MOESM1_ESM.pdf]

Description of Additional Supplementary Files

File name: Supplementary Data 1

Description: Data of Fig. 2a for downstream/upstream BERs at different received MMW/sub-6 GHz powers.

File name: Supplementary Data 2

Description: Data of Fig. 2b for downstream/upstream EVMs at different received MMW/sub-6 GHz powers.

File name: Supplementary Data 3

Description: Data of Fig. 4a for the subcarrier EVMs of 10-Gbps/26-GHz (x-polarisation; upstream) and 10-Gbps/38-GHz (y-polarisation; downstream) 16-QAM-OFDM signals at different subcarrier indices.

File name: Supplementary Data 4

Description: Data of Fig. 4b for the subcarrier EVMs of 1-Gbps/3.7-GHz (x-polarisation; upstream) and 1-Gbps/4.4-GHz (y-polarisation; downstream) 16-QAM-OFDM signals at different subcarrier indices.
